# Total cost of coverage for members in California's marketplace

**DOI:** 10.1093/haschl/qxaf135

**Published:** 2025-07-09

**Authors:** Emily Kohn, Emory Wolf, Isaac Menashe, Katie Ravel

**Affiliations:** Covered California, Sacramento, CA, United States; Covered California, Sacramento, CA, United States; Covered California, Sacramento, CA, United States; Covered California, Sacramento, CA, United States

**Keywords:** cost of coverage, Affordable Care Act, American Rescue Plan, state-based marketplace, advanced premium tax credit

## Abstract

**Introduction:**

Discussions of coverage affordability within the Affordable Care Act (ACA) marketplaces generally focus on premium costs. However, the total cost of coverage includes out-of-pocket expenses such as copays and deductibles.

**Method:**

Using claims and enrollment data from Covered California, California's ACA marketplace, we document the total cost of coverage for households enrolled in full-year subsidized marketplace coverage as a percent of household income in 2019 and 2022.

**Results:**

In 2022, the average total cost of coverage for households below 400% of the federal poverty line was $2,519, representing 6.6% of household income. In 2019, the average cost of coverage for this population was 8.7%. The decrease was driven by decreases in net premiums, as out-of-pocket costs remained relatively unchanged. We also show that the total cost of coverage as a percent of household income is progressively distributed and varies substantially by plan metal tier.

**Conclusion:**

Overall, our findings indicate that income-adjusted affordability measures established by the ACA have reduced financial burdens for many consumers.

## Teaser

Using claims and enrollment data from Covered California, California's ACA marketplace, we document the total cost of coverage, including both premiums and out-of-pocket costs, for households enrolled in full-year subsidized marketplace coverage as a percent of household income. Our findings indicate that income-adjusted affordability measures established by the ACA have reduced financial burdens for many consumers.

## Introduction

Rising costs of health insurance coverage are a growing concern. The average employee contribution for an employer-sponsored family plan increased by 22% since 2017.^1^ Out-of-pocket costs (including copays, coinsurance, and deductibles) are also increasing: the average deductible for an employer-sponsored plan as a percent of median income rose from 3.3% in 2010 to 4.7% in 2020. During the same time period, the cost of premium contributions and deductible for employer-sponsored plans increased from 9% to 12% of the median income.^2^ Together, these rising costs place a substantial financial burden on the US population, potentially exacerbating socioeconomic disparities.^3^ Half of adult Americans say it is difficult to afford the cost of health coverage, and 1 in 4 has delayed needed care due to the cost in the last 12 months.^4,5^

To make access to coverage more affordable and equitable, the Affordable Care Act (ACA) implemented the marketplace's income-adjusted premium tax credit (or the Advanced Premium Tax Credit [APTC]). The premium tax credit reduces enrollees' premiums based on household income as a percent of the federal poverty level (FPL). Premium tax credits are an evolving policy instrument: in March 2021, Congress increased premium tax credit amounts for all income brackets and expanded eligibility to middle-income households as part of the American Rescue Plan Act (ARPA). These enhanced premium tax credits were subsequently extended through the 2025 plan year through the Inflation Reduction Act of 2022. Marketplace enrollment increased substantially after the passage of the American Rescue Plan. Covered California, the ACA marketplace for California, saw enrollment increase from 1.2 million enrollees in December 2019 to 1.6 million in December 2022,^6^ covering approximately 4.4% of insured Californians.^7^

However, households also face expenses when they access care,^8^ and these costs can be a substantial part of the total cost of coverage. In 2022, 23% of nonelderly adults were found underinsured, meaning that out-of-pocket or deductible costs took up a high percentage of household income.^9^ Plans available through the ACA marketplaces conform to metal tiers based on actuarial value (AV), ranging from Bronze (60% AV) to Platinum (90% AC). In general, plans with lower AV cover a lower share of total costs of care and have lower premiums. To improve affordability of out-of-pocket costs, the ACA marketplaces provide cost sharing reduction (CSR) Silver plans, which offer richer benefits but have the same premium cost as standard Silver plans, for households with incomes below 250% of FPL. However, Covered California is unusual among marketplaces in having a standard benefit design that applies to all plans.

While the impact of premium tax credits on premium affordability is well-documented,^10^ little evidence exists on the overall burden of out-of-pocket costs incurred in marketplace plans, an essential aspect of understanding the total cost of coverage for households with ACA marketplaces. This study leverages a unique data set containing detailed information on household income, premiums, and out-of-pocket costs to analyze the total cost of coverage for marketplace enrollees in California and to assess trends in affordability between 2019 and 2022.^11^

## Data and methods

Our analysis combines administrative enrollment and claims data for Covered California members enrolled in 2019 and 2022. We include 2 years of data to report how total cost of coverage varies with and without the availability of enhanced premium tax credits, which are set to expire after 2025. Administrative data include projected household income, household size, and premium amounts for each household. Claims data include all medical claims for marketplace plans. Our analysis does not include costs outside of Covered California medical coverage, such as dental claims and records for household members not enrolled with Covered California.

We sum annual household net-of-tax credit premiums and out-of-pocket costs to calculate a total cost of coverage estimate. To assess how this relates to a household's finances, we calculate the total cost of coverage as a percent of household income, which we use as our primary measure. To maintain comparability across households, households that did not apply for premium tax credits (Households with incomes above 400% of FPL in 2019 are still included in the data because they submitted applications for subsidized coverage but were above the cutoff for premium tax credit eligibility.) and households with less than 12 months of enrollment in the plan year are excluded. These restrictions reduce our study population by approximately 47%, or approximately 29% of member-months (see Table S1 for more information) Prior work has shown that enrollment length varies by demographic group, so results should not necessarily be considered representative for households with shorter enrollment tenures.^12^

In general, demographic distributions remained consistent across the years, with the exception of the number of households with incomes greater than 400% of FPL that submitted applications for subsidized coverage. In 2019, these households were not eligible for premium tax credits, but they gained eligibility following the ARPA's premium tax credit expansion in 2021. This group comprises approximately 5% of the 2019 sample but 10% of the 2022 sample. Due to the sizeable decrease in total cost of coverage due to introduction of premium tax credits for this group in 2022, we present all results for the over 400% FPL population separately from the under 400% FPL population.

## Results

**Figure 1.**
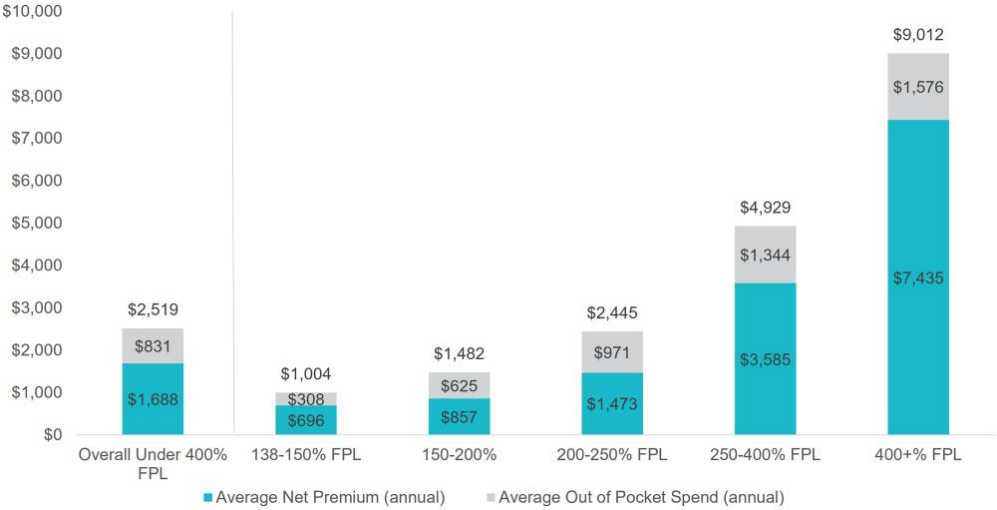
2022 cost of coverage in dollars by federal poverty level (FPL) bracket. Authors' analysis of 2022 administrative enrollment and claims data for Covered California members.

As shown in Figure 1, a household with income under 400% FPL spent, on average, $2519 on coverage over the course of 2022, while a household with income over 400% FPL spent an average of $9012. The average cost of coverage for the full population was $3112 (not shown in graph). Overall, net premiums were the primary driver of costs of coverage for all income groups. As costs increase with income, the overall distribution of the total cost of coverage is roughly progressive.

**Figure 2.**
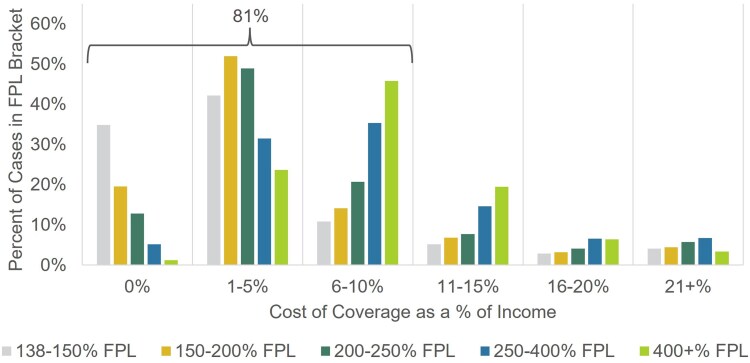
Distribution of 2022 cost of coverage as a percent of income by federal poverty level (FPL) bracket. Authors' analysis of 2022 administrative enrollment and claims data for Covered California members.

To better understand how costs relate to overall financial picture of a household, we calculate total cost of coverage as a percent of household income. The distribution of this measure shows wide variation (Figure 2), even when stratified by household income bracket. While the average cost of coverage was 6.5% of household income, over 80% of households spend less than 10% of their income on coverage. However, across all income brackets, some households continue to spend a high percentage of their income on coverage. Approximately 3% to 6% of each income bracket spends over 20% of their household income on coverage; these are generally households with high out-of-pocket spending on catastrophic events, such as hospitalizations. For a low-income household, such as an individual earning 150% of the FPL, the maximum out-of-pocket amount for a Bronze plan ($8200)^8^ could represent up to 43% of their income for the year.

Much of the variation in total cost of coverage is expected to be driven by plan choice, as members enrolled in plans with higher actuarial value (AV), by design, will pay more upfront in monthly premiums to protect against the risk of higher health care costs when care is used.

**Figure 3.**
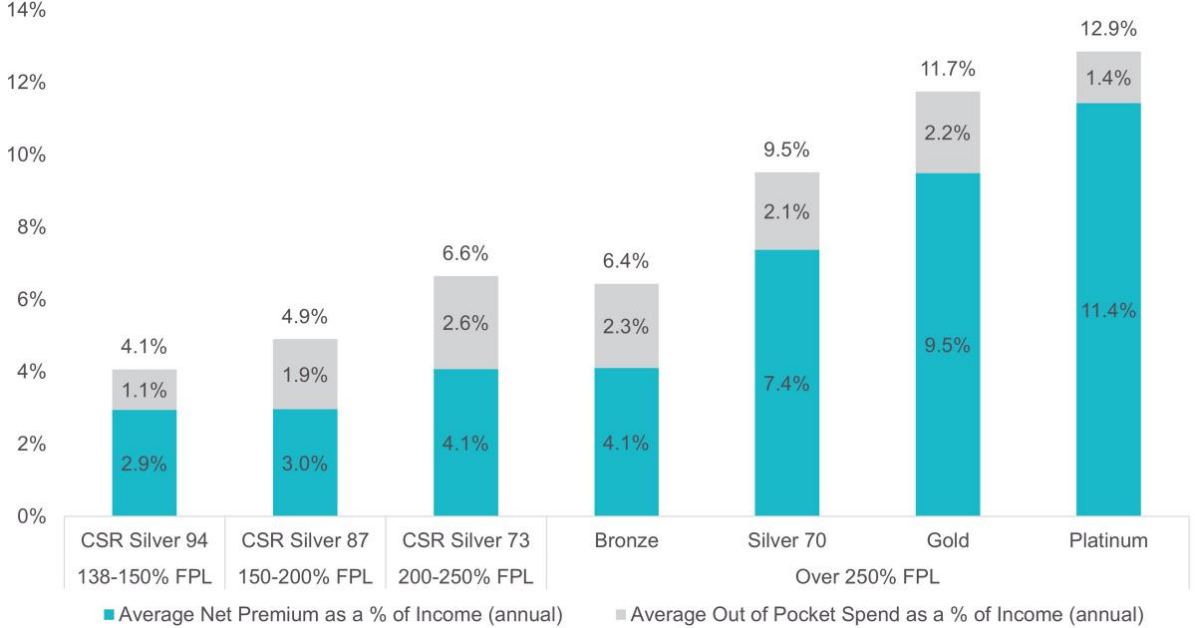
2022 cost of coverage as a percent of income by metal tier. Authors' analysis of 2022 administrative enrollment and claims data for Covered California members.

As expected, we find that total cost of coverage varies with AV (Figure 3). With substantial premium tax credits, lower income consumers in CSR plans (especially Silver 94 and 87) faced the lowest total cost of coverage as a percentage of income. Out-of-pocket costs as a share of income were lowest for those in the highest AV plans. Members in Gold and Platinum plans generally have higher total allowed amounts for services than members in lower AV plans, suggesting that these members anticipate utilizing more health care and therefore chose to pay higher premiums to access lower cost sharing plans (see Figure S1 for more details.) Even with sorting among metal tiers, differences in total cost of coverage are driven by net premiums, as out-of-pocket costs remain around 2%-3% of income for most metal tiers. (For the numbers underlying Figure 3 and the distribution of out-of-pocket costs as a percent of income, see Tables S2 and S3.)

**Figure 4.**
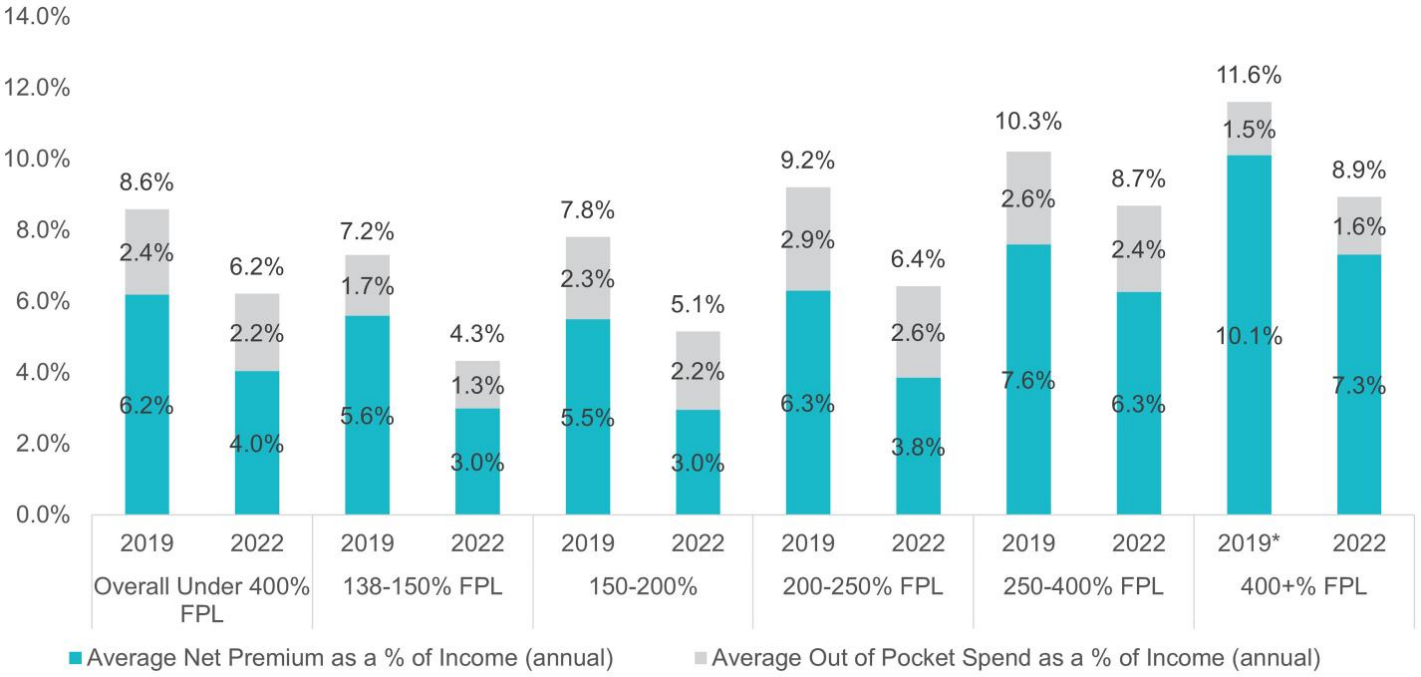
2019 and 2022 cost of coverage as a percent of income by income bracket. Authors' analysis of 2019 and 2022 administrative enrollment and claims data for Covered California members. Cases with over 400% federal poverty level (FPL) could not receive subsidies in 2019. We include gross premiums as a percent of income for all cases with over 400% FPL in 2019.

From 2019 to 2022, households across all income groups in California's ACA marketplace saw sizeable decreases in the total cost of coverage as a percent of income (Figure 4). For households with incomes below 400% of FPL, costs fell from 8.6% to 6.2% and from 11.6% to 8.9% for households with incomes above 400% of FPL. The average total cost of coverage as a percent of income dropped below 10% for all income brackets in 2022.

## Discussion and conclusion

With income-based affordability support, the average total cost of coverage—including both premiums and out-of-pocket costs—for households across all income brackets in California's ACA marketplace was below 10% of income in 2022. This includes middle-income households who were previously ineligible for premium tax credits prior to the passage of ARPA in 2021. Comparable measures for employer-sponsored coverage show that the average cost of premiums and deductibles increased to 11.6% of median household income between 2015 and 2020.^2^

The total cost of coverage was strongly progressive, with lower income households paying less as a percentage of income for coverage than higher income households. This was driven by net premiums and the progressivity of premium tax credits; out-of-pocket costs as a percent of income are relatively similar across all income brackets. However, our findings underscore that some consumers may still face uneven financial burdens, including costs that exceed 10% of income.

As policymakers consider changes to programs supporting affordability in a time of rising health care costs, both the total cost of coverage and care measure and the measure of financial burden as a share of income used in this analysis could be a model for monitoring impacts.^13^ Overall, our findings indicate that income-adjusted affordability measures established by the ACA and extended by ARPA have reduced financial burdens for many consumers. Further research is needed to study the drivers of the variation in total cost of coverage, including chronic conditions and other markers of health status, and to connect these costs with other financial burdens faced by households across the income spectrum.

## Supplementary Material

qxaf135_Supplementary_Data
