# Supplementary material for: Total cost of coverage for members in California's marketplace
Source: Health Aff Sch. 2025 Jul 9;3(8):qxaf135. doi: 10.1093/haschl/qxaf135 (PMC12322486; doi:10.1093/haschl/qxaf135)
Supplement: qxaf135_Supplementary_Data [file qxaf135_supplementary_data.zip › Health Affairs Scholar Supplementary Tables (1).docx]

**Supplementary Table 1: Overall Count of Households and Count of Households Enrolled for Twelve Months in 2022**

|  | **Count of Households** | **Count of Households Enrolled for 12 Months** | **Percent of Households Enrolled for 12 Months** | **Member Months for All Subsidized Households** | **Member Months for Households Enrolled for 12 Months** | **Percent of Member Months with Households Enrolled for 12 Months** |
| --- | --- | --- | --- | --- | --- | --- |
| **Total** | 1,468,374 | 777,632 | 53% | 18,628,890 | 13,304,532 | 71% |
| **Metal Tier** |  |  |  |  |  |  |
| Bronze | 382,426 | 199,381 | 52% | 4,815,982 | 3,471,564 | 72% |
| Silver 70 | 206,974 | 104,567 | 51% | 3,069,927 | 2,164,824 | 71% |
| Silver 73 | 138,417 | 74,278 | 54% | 1,685,515 | 1,212,948 | 72% |
| Silver 87 | 330,001 | 186,717 | 57% | 4,011,895 | 2,935,440 | 73% |
| Silver 94 | 213,382 | 117,009 | 55% | 2,485,881 | 1,763,868 | 71% |
| Gold | 133,009 | 65,438 | 49% | 1,789,138 | 1,234,212 | 69% |
| Platinum | 64,165 | 30,242 | 47% | 770,552 | 521,676 | 68% |
| **FPL Bracket** |  |  |  |  |  |  |
| 138% to 150% FPL | 241,771 | 128,458 | 53% | 2,747,199 | 1,923,324 | 70% |
| 150% to 200% FPL | 438,559 | 247,240 | 56% | 5,186,279 | 3,810,432 | 73% |
| 200% to 250% FPL | 264,911 | 139,467 | 53% | 3,095,830 | 2,213,388 | 71% |
| 250% to 400% FPL | 371,138 | 191,374 | 52% | 5,503,378 | 3,932,328 | 71% |
| 400+% FPL | 151,995 | 71,093 | 47% | 2,096,204 | 1,425,060 | 68% |

**Supplementary Table 2: Distribution of Out-of-Pocket Costs as a Percent of Income**

|  | **138-150% FPL** | **150-200% FPL** | **200-250% FPL** | **250-400% FPL** | **400+% FPL** | **Overall** |
| --- | --- | --- | --- | --- | --- | --- |
| **0%^[[1]](#endnote-1)^** | 92,669 (72.1%) | 151,574 (61.3%) | 79,292 (56.8%) | 106,938 (55.8%) | 45,680 (64.2%) | 476,153 (61.2%) |
| **1-5%** | 31,658 (24.6%) | 70,837 (28.7%) | 44,533 (31.9%) | 63,254 (33.1%) | 20,220 (28.4%) | 230,502 (29.6%) |
| **6-10%** | 2,271 (1.8%) | 13,111 (5.3%) | 6,871 (4.9%) | 10,282 (5.4%) | 3,685 (5.2%) | 36,220 (4.7%) |
| **11-15%** | 658 (0.5%) | 6,962 (2.8%) | 3,447 (2.5%) | 5,464 (2.9%) | 1,214 (1.7%) | 17,745 (2.3%) |
| **16-20%** | 295 (0.2%) | 1,694 (0.7%) | 2,413 (1.7%) | 3,252 (1.7%) | 178 (0.3%) | 7,832 (1%) |
| **Over 20%** | 907 (0.7%) | 3,062 (1.2%) | 2,911 (2.1%) | 2,184 (1.1%) | 116 (0.2%) | 9,180 (1.2%) |
| **Total** | **128,458 (100%)** | **247,240 (100%)** | **139,467 (100%)** | **191,374 (100%)** | **71,093 (100%)** | **777,632 (100%)** |

**Supplementary Table 3: Distribution of Total Cost of Coverage as a Percent of Income**

|  | **138-150% FPL** | **150-200% FPL** | **200-250% FPL** | **250-400% FPL** | **400+% FPL** | **Overall** |
| --- | --- | --- | --- | --- | --- | --- |
| **0%^1^** | 44,783 (34.9%) | 48,321 (19.5%) | 17,935 (12.9%) | 9,940 (5.2%) | 881 (1.2%) | 121,860 (15.7%) |
| **1-5%** | 54,171 (42.2%) | 128,424 (51.9%) | 68,190 (48.9%) | 60,326 (31.5%) | 16,819 (23.7%) | 327,930 (42.2%) |
| **6-10%** | 13,952 (10.9%) | 34,985 (14.2%) | 28,905 (20.7%) | 67,667 (35.4%) | 32,593 (45.8%) | 178,102 (22.9%) |
| **11-15%** | 6,628 (5.2%) | 16,770 (6.8%) | 10,735 (7.7%) | 27,921 (14.6%) | 13,833 (19.5%) | 75,887 (9.8%) |
| **16-20%** | 3,665 (2.9%) | 7,821 (3.2%) | 5,681 (4.1%) | 12,577 (6.6%) | 4,551 (6.4%) | 34,295 (4.4%) |
| **Over 20%** | 5,259 (4.1%) | 10,919 (4.4%) | 8,021 (5.8%) | 12,943 (6.8%) | 2,416 (3.4%) | 39,558 (5.1%) |
| **Total** | **128,458 (100%)** | **247,240 (100%)** | **139,467 (100%)** | **191,374 (100%)** | **71,093 (100%)** | **777,632 (100%)** |

1. Note that a household that spends between 0.0% and 0.5% of income on out-of-pocket or total coverage will be included in this group; this chart should not be read as showing that 61.2% of Covered California households spend $0 on out-of-pocket costs. For reference, the Covered California Plan Performance report for 2022 shows that 12% of the population utilized no healthcare in 2022. For more details, see 2024 Plan Performance Report: Healthcare Evidence Initiative (HEI) Data [Internet]. Sacramento: Covered California [Cited 2025 June 9] [https://hbex.coveredca.com/data-research/plan-performance-reports/2024/Release Year 2024 PPR - HEI Measures.pdf](https://hbex.coveredca.com/data-research/plan-performance-reports/2024/Release%20Year%202024%20PPR%20-%20HEI%20Measures.pdf). [↑](#endnote-ref-1)
