# Supplementary figures and images for: Total cost of coverage for members in California's marketplace
Source: Health Aff Sch. 2025 Jul 9;3(8):qxaf135. doi: 10.1093/haschl/qxaf135 (PMC12322486; doi:10.1093/haschl/qxaf135)

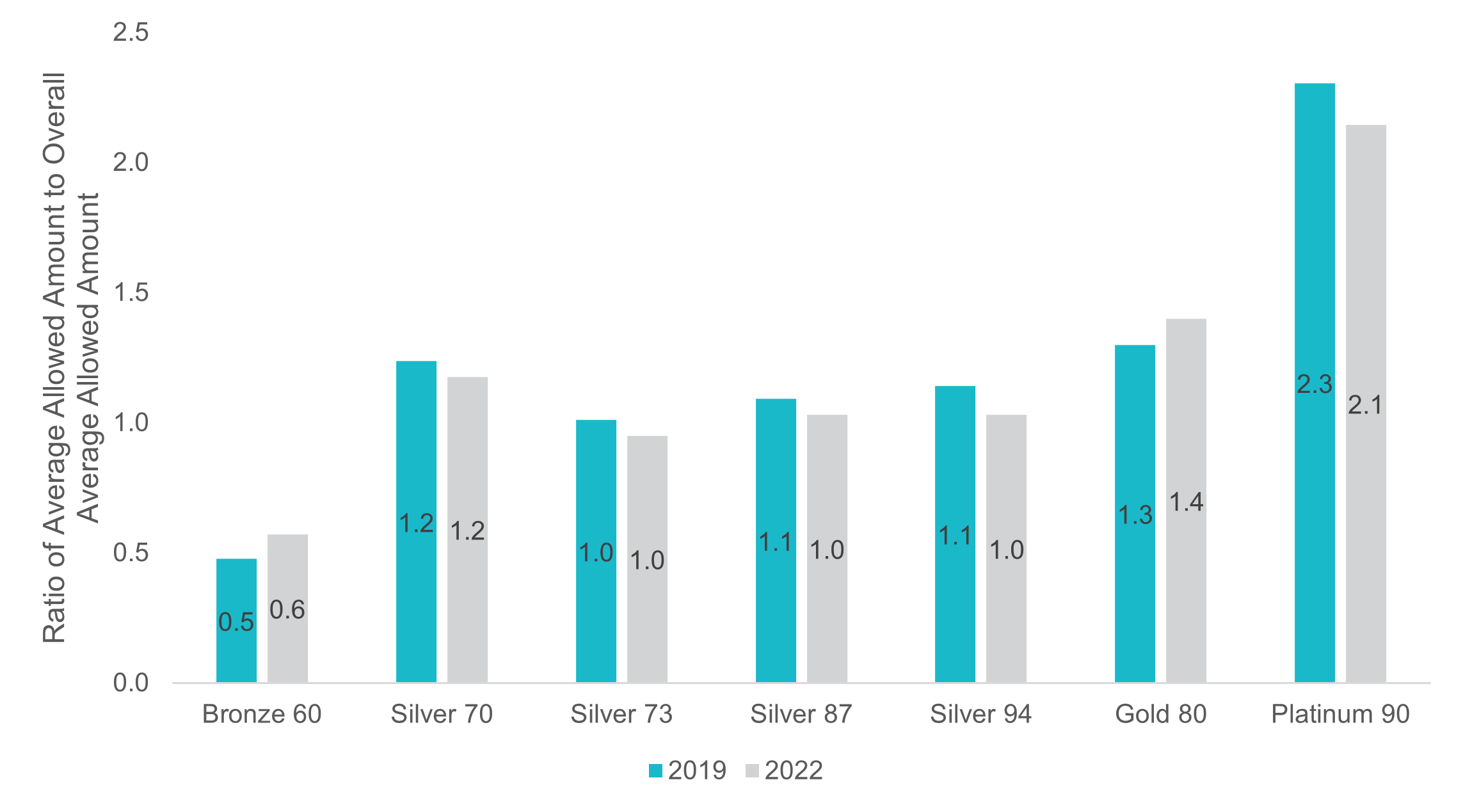

Supplement: qxaf135_Supplementary_Data [file qxaf135_supplementary_data.zip › Supplementary Figure 1.png]
